# Value of Preoperative Modified Body Mass Index in Predicting Postoperative 1-Year Mortality

**DOI:** 10.1038/s41598-018-22886-6

**Published:** 2018-03-15

**Authors:** Tak Kyu Oh, Jaebong Lee, Jung-Won Hwang, Sang-Hwan Do, Young-Tae Jeon, Jin Hee Kim, Kooknam Kim, In-Ae Song

**Affiliations:** 1 Interdepartment of Critical Care Medicine, Seoul National University Bundang Hospital, 82, Gumi-ro 173 Beon-gil, Bundang-gu, Seongnam-si, Gyeonggi-do 463-707 Korea; 2 Department of Anesthesiology and Pain Medicine, Seoul National University Bundang Hospital, 82, Gumi-ro 173 Beon-gil, Bundang-gu, Seongnam-si, Gyeonggi-do 463-707 Korea; 3 Medical Research Collaborating Center, Seoul National University Bundang Hospital, 82, Gumi-ro 173 Beon-gil, Bundang-gu, Seongnam-si, Gyeonggi-do 463-707 Korea

## Abstract

Serum albumin and conventional BMI (cBMI) are commonly used indices of malnutrition status. Because cBMI does not reflect fluid accumulation, modified body mass index (mBMI, serum albumin × cBMI) is a more accurate measure of malnutrition status. This study investigated the association between preoperative mBMI and postoperative 1-year mortality, in comparison with serum albumin and cBMI. Medical records of 80,969 adult patients who underwent surgical procedures in a tertiary care hospital between 1 January, 2011 and 31 December, 2015 were retrospectively reviewed. Postoperative 1-year mortality increased with reduction in cBMI, mBMI, and albumin separately (*P* < 0.001). When considering interaction between cBMI and albumin, albumin was the only significant factor of postoperative 1-year mortality [odds ratio: 0.377, 95% confidence interval (0.245–0.579), *P* < 0.001], while cBMI or interaction (cBMI * albumin) was not significant (*P* > 0.05). Adjusted area under the curve (AUC) was highest (0.885) in the overall model (cBMI + albumin + cBMI * albumin); adjusted AUC between mBMI and albumin did not differ significantly (*P* = 0.558). Low albumin is the strongest independent predictor of postoperative 1-year mortality among the three variables (albumin, cBMI, mBMI). Adding cBMI to albumin does not increase the validity of the AUC of albumin.

## Introduction

Malnutrition, a common complication of chronic or severe disease, is known to exacerbate disease prognoses^1^. Patients’ malnutrition status may have adverse effects on their postoperative mortality or prognosis^2^. Therefore, preoperative malnutrition is an important clinical problem; conventional body mass index (cBMI) and serum albumin level are commonly used as indicators of malnutrition status^3,4^. Recent studies have reported that low preoperative BMI^5,6^ and low serum albumin level^7,8^ exacerbate postoperative mortality and prognosis. This indicates the importance of preoperative nutritional status given the recent trend of increasing number of surgeries among elderly patients^9^.

However, it is still not clear how accurately cBMI measures the nutritional status of patients^10,11^, because it has limitations in reflecting fluid balance, such as fluid accumulation or dehydration. In fact, such limitations of cBMI were suggested to affect patients undergoing liver transplantation, in which ascites is a common problem. A new approach suggested to overcome these limitations, is the use of the modified BMI (mBMI)^12,13^. The mBMI considers serum albumin level and cBMI in combination, and thus is a relevant tool for patients who undergo liver transplantation^10–13^. However, there are no data on the effects of mBMI on the postoperative prognosis in the general population.

Therefore, we aimed to investigate the value of preoperative mBMI as a predictor of postoperative mortality, as compared to previously used predictors (cBMI and serum and albumin).

## Results

**Figure 1:**
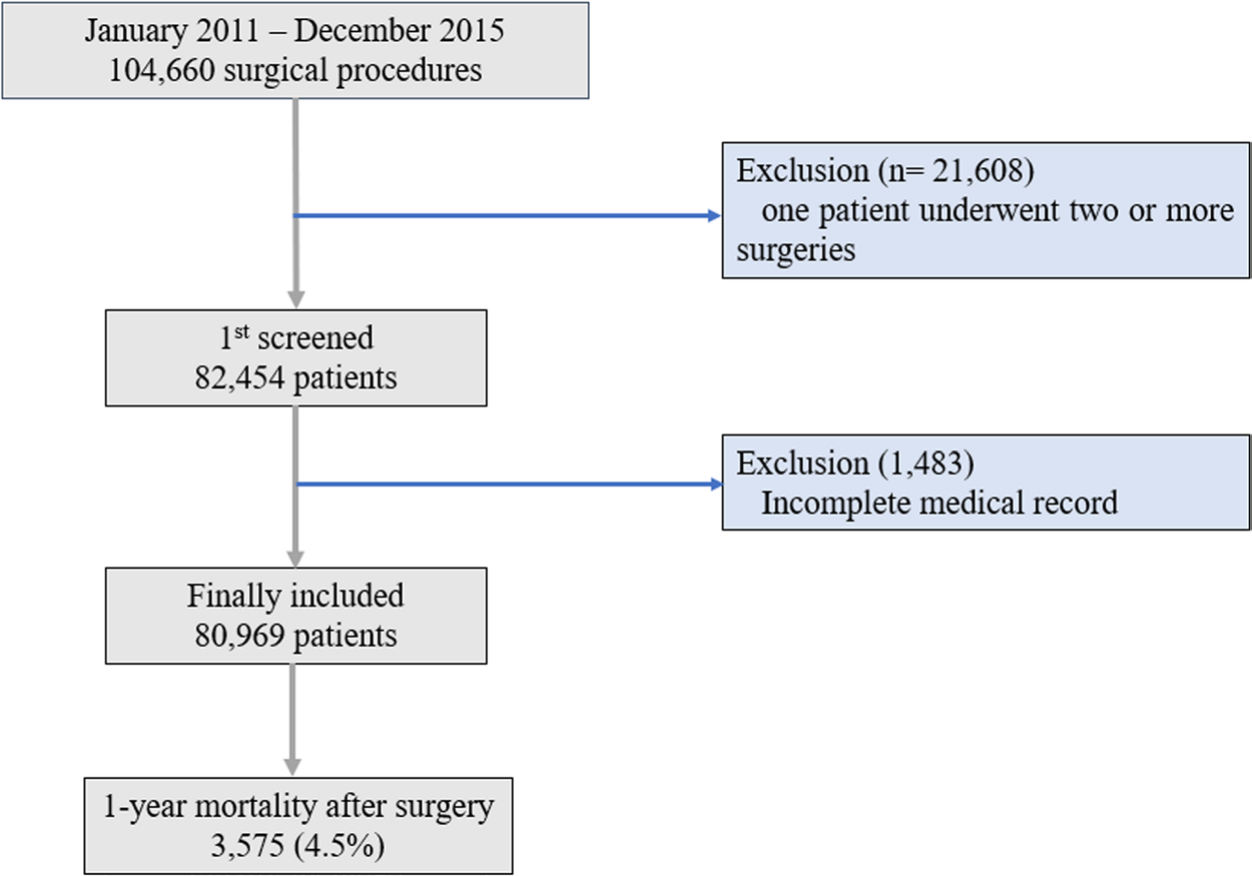
Flow chart for patient selection.

A total of 82,452 adult patients underwent 104,660 cases of operations or surgical procedures at Seoul National University Bundang Hospital (SNUBH) between January 2011 and December 2015. Of these, 1,483 patients were excluded due to inaccurate or incomplete medical records; the remaining 80,969 patients were included in the final analysis. Among the 80,969 patients, 3,575 (4.4%) died within 1 year of the operation (Fig. 1). The demographic and clinical characteristics of the final analysis are presented in Table 1.

**Table 1:** Baseline characteristics of all patients.

Total (n = 80,969)	Number (%)	Mean	SD
Operation			
Cardiovascular surgery	1,134 (1.4%)		
Non-cardiovascular surgery	79,835 (98.6%)		
Male (%)	35,302 (43.6%)		
Age (year)		54.5	16.2
cBMI (kg/m^2^)		24.1	3.6
Preoperative Laboratory Test Result			
Hemoglobin (g/dL)		13.5	1.9
WBC (x1000/uL)		6.7	2.4
Platelet (x1000/uL)		246.3	71.0
Prothrombin time (INR)		1.1	0.1
Aspartate aminotransferase (IU/L)		24.6	104.0
Alanine aminotransferase (IU/L)		24.4	41.6
Albumin (g/dL)		4.3	0.5
Glucose (mg/dL)		109.0	35.9
aPTT (sec)		36.7	5.9
Blood urea nitrogen (mg/dL)		14.7	7.4
Serum creatinine (mg/dL)		0.9	0.8
Serum sodium (mmol/L)		140.4	2.8
Serum potassium (mmol/L)		4.2	0.4
Cormack grade			
I,II	67,771 (83.7%)		
III, IV	13,198 (16.3%)		
Preoperative Comorbidity			
Diabetes mellitus	9,635 (11.9%)		
Hypertension	20,404 (25.2%)		
Ischemic heart disease	4,534 (5.6%)		
Neurologic disease	3,400 (4.2%)		
ASA classification			
I	36,921 (45.6%)		
II	39,350 (48.6%)		
III	4,534 (5.6%)		
IV, V, VI	162 (0.2%)		
Type of anesthesia			
History of general anesthesia	31,578 (39.0%)		
General anesthesia	54, 897 (67.8%)		
Regional anesthesia	10,607 (13.1%)		
Monitored anesthesia care	14,103 (17.4%)		
Local anesthesia	1,281 (1.6%)		
Postoperative ICU admission	3.7%		

SD, standard deviation; cBMI, conventional body mass index; WBC, white blood cell; ASA, American Society of Anesthesiologists; aPTT, activated partial thromboplastin time; ICU, intensive care unit.

### One-year mortality with respect to preoperative cBMI, mBMI, and albumin

**Figure 2:**
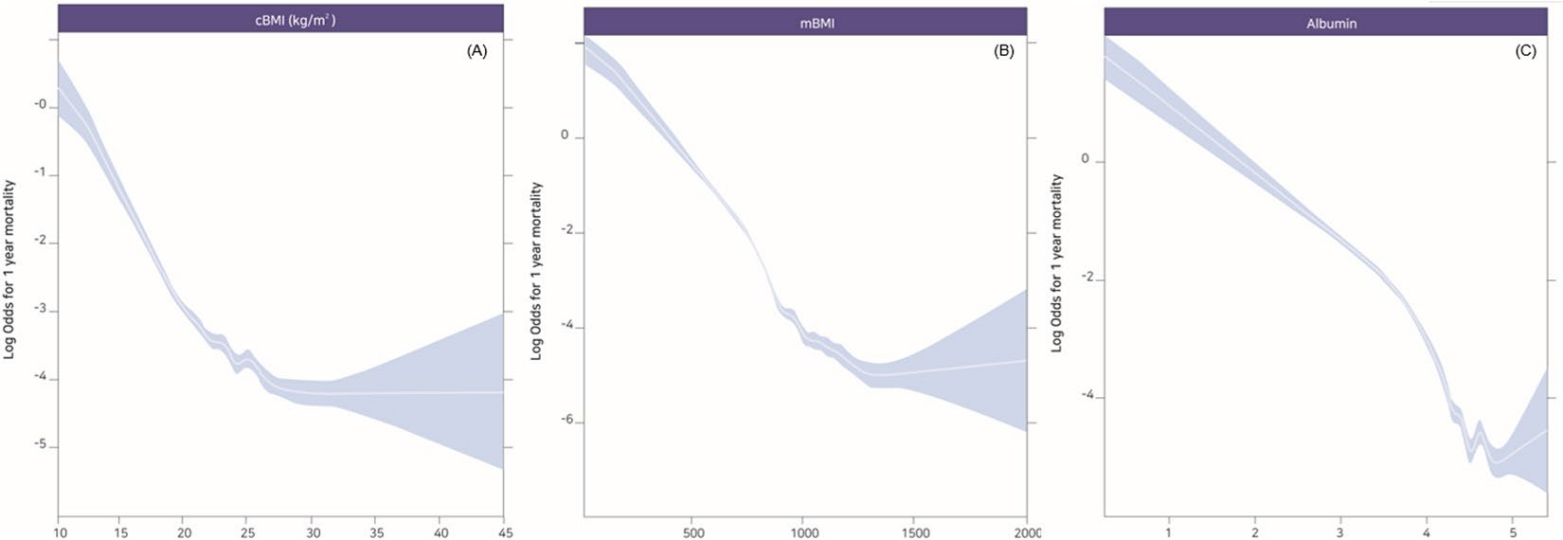
Log odds for postoperative 1-year mortality plotted against the changes of preoperative cBMI (**A**), mBMI (**B**), and albumin (**C**).

The (log) ORs of postoperative 1-year mortality were plotted against changes in preoperative cBMI, mBMI, and albumin (Fig. 2A–C). Table 2 shows the univariate logistic regression analysis for postoperative 1-year mortality in all patients. Supplemental Table 1 shows the results of individual multivariate logistic regression model without considering the interaction between cBMI and albumin. Lower cBMI (0.847, 95% confidence interval [CI]: 0.836–0.858), mBMI (0.995, 95% CI: 0.995–0.995), and albumin (0.230, 95% CI: 0.214–0.247) were associated with increased postoperative 1-year mortality separately. However, in the multivariate model as shown in Table 3, when interaction among the three variables were considered, only albumin was significantly associated with postoperative 1-year mortality [OR: 0.377, 95% CI (0.245–0.579), *P* < 0.001], whereas interaction (cBMI * albumin) and cBMI showed no significant association with postoperative 1-year mortality (*P* > 0.05).

**Table 2:** Univariate logistic regression analysis for 1-year mortality in all patients.

Variable	1-year death (n = 3,575)	*P*-value	Odds ratio (95% CI)	*P*-value
Operation		<0.001		
Non-cardiovascular surgery	3,428 (95.9%)		Ref	
Cardiovascular surgery	147 (4.1%)		3.14 (2.56–3.86)	<0.001
Gender		<0.001		
Male	2,252 (63.0%)		Ref	
Female	1,323 (37.0%)		0.44 (0.41–0.48)	<0.001
Age (yr), mean (SD)	67.5 (13.9)	<0.001	1.06 (1.06–1.07)	<0.001
cBMI (kg/m^2^), mean (SD)	22.09 (3.70)	<0.001	0.83 (0.82–0.84)	<0.001
<18.5	575 (16.1%)		Ref	
18.5–24.9	2,266 (63.4%)		0.25 (0.23–0.28)	<0.001
25–29.9	661 (18.5%)		0.14 (0.12–0.16)	<0.001
≥30	73 (2.0%)		0.09 (0.07–0.12)	<0.001
mBMI, mean (SD)	805.9 (223.7)	<0.001	0.99 (0.99–0.99)	<0.001
Albumin (g/dL), mean (SD)	3.6 (0.7)	<0.001		
<2.5	182 (5.1%)		Ref	
2.5–3.5	1,312 (36.7%)		0.31 (0.25–0.39)	<0.001
>3.5	2,081 (58.2%)		0.03 (0.03–0.04)	<0.001
Diabetes mellitus	21.5%	<0.001	2.09 (1.90–2.30)	<0.001
Hypertension	33.3%	<0.001	1.51 (1.39–1.64)	<0.001
Ischemic heart disease	14.3%	<0.001	2.96 (2.65–3.31)	<0.001
Neurologic disease	10.0%	<0.001	2.65 (2.33–3.02)	<0.001
ASA classification				
I	382 (10.7%)		Ref	
II	2,066 (57.8%)		5.23 (4.61–5.94)	<0.001
III	1,047 (29.3%)		26.23 (23.43–30.94)	<0.001
IV	58 (1.9%)		72.76 (50.77–104.28)	<0.001
V	12 (0.3%)		31.83 (8.94–113.40)	<0.001
VI	10 (0.3%)		79.58 (25.88–244.72)	<0.001
History of general anesthesia	2,045 (57.2%)	<0.001	2.15 (1.99–2.32)	<0.001
Type of anesthesia		<0.001		
General anesthesia	2,313 (64.7%)		Ref	
Regional anesthesia	311 (8.7%)		0.69 (0.60–0.79)	<0.001
Monitored anesthesia care	944 (26.4%)		1.47 (1.35–1.61)	<0.001
Local anesthesia	8 (0.2%)		8.99 (3.31–24.40)	<0.001
Postoperative ICU admission	626 (17.5%)	<0.001	6.38 (5.73–7.10)	<0.001

SD, standard deviation; cBMI, conventional body mass index; mBMI, modified body mass index; ASA, American Society of Anesthesiologists; ICU, intensive care unit.

**Table 3:** Multivariate logistic regression analysis for 1-year mortality regarding three preoperative variables (cBMI, mBMI, and albumin).

Variable	Multivariate model	*P*-value
	Odds Ratio (95% CI)	
Type of operation		
Non-cardiovascular surgery	Ref	
Cardiovascular surgery	0.674 (0.526–0.857)	0.002
Gender		
Male	Ref	
Female	0.547 (0.50–0.60)	<0.001
Age (year)	1.03 (1.03–1.04)	<0.001
Diabetes mellitus	1.03 (0.92–1.15)	0.621
Hypertension	0.71 (0.64–0.78)	<0.001
Ischemic Heart Disease	0.86 (0.74–0.99)	0.035
Neurologic Disease	0.97 (0.83–1.14)	0.738
ASA class		
I	Ref	
II	2.83 (2.44–3.29)	<0.001
III	5.58 (4.66–6.70)	<0.001
IV, V, VI	11.37 (7.43–17.26)	<0.001
History of general anesthesia	Ref (No)	
	1.228 (1.17–1.40)	<0.001
Type of anesthesia		
General anesthesia	Ref	
Regional anesthesia	0.53 (0.45–0.61)	<0.001
Monitored anesthesia care	1.09 (0.98–1.22)	0.100
Local anesthesia	2.80 (0.70–10.10)	0.131
Postoperative ICU admission	Ref (No)	
	1.64 (1.42–1.90)	<0.001
cBMI (kg/m^2^)	0.94 (0.88–1.01)	0.087
Interaction (cBMI * Albumin)	0.98 (0.97–1.00)	0.076
Albumin	0.38 (0.25–0.58)	<0.001

cBMI, conventional body mass index; mBMI, modified body mass index; IHD, ischemic heart disease; NUD, neurologic disease; ASA, American Society of Anesthesiologists; ICU, intensive care unit.

### Comparison of preoperative cBMI, mBMI, and albumin according to ROC curve

**Figure 3:**
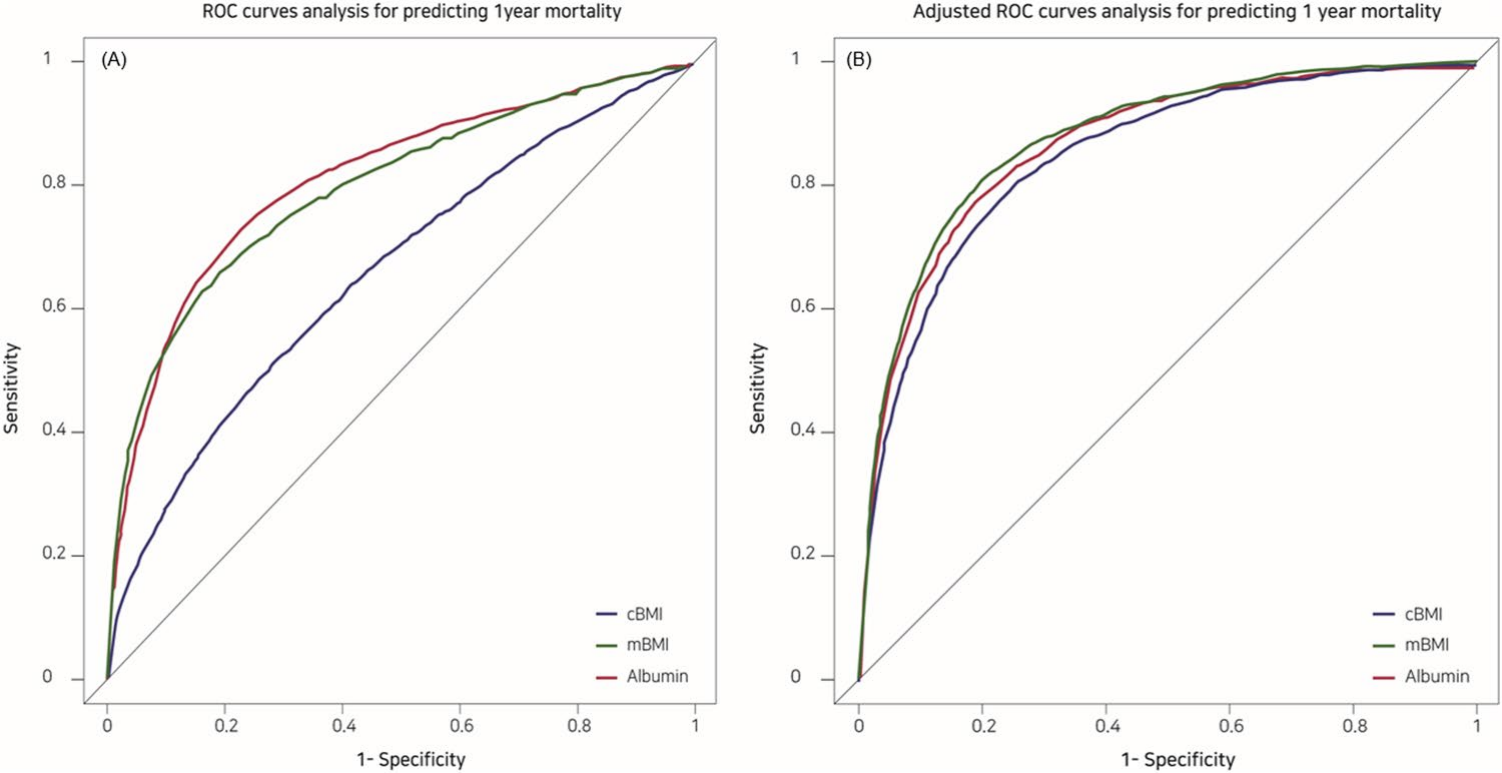
ROC curves (**A**) and covariate-adjusted ROC curves (**B**) showing the risk of postoperative 1-year mortality with respect to preoperative cBMI, mBMI, and albumin.

Figure 3A presents the ROC curves that show the risk for postoperative 1-year mortality with respect to preoperative cBMI, mBMI, and albumin. Figure 3B presents the ROC curves that predict the risk for postoperative 1-year mortality with respect to the preoperative cBMI, mBMI, and albumin after adjusting for the covariates (type of operation, gender, age, history of diabetes mellitus, hypertension, and ischaemic heart disease, ASA classification, history of general anesthesia, type of anesthesia, postoperative ICU admission). Prior to adjusting for the covariates, the AUC were cBMI Model = 0.659, mBMI Model = 0.797, albumin Model = 0.811, and overall Model = 0.820 (cBMI + albumin + cBMI * albumin); Table 4. After adjustment for the covariates, AUC was highest with the overall Model [cBMI + albumin + cBMI * albumin; 0.885, 95% CI (0.878–0.891)], followed by mBMI [0.879, 95% CI (0.873–0.886)], albumin [0.878, 95% CI (0.872–0.885)], and cBMI [0.853, 95% CI (0.846–0.860)]; Table 4. In addition, covariate adjusted AUC of mBMI was higher than cBMI (*P* < 0.001), while covariate adjusted AUC of mBMI and albumin was not significantly different in DeLong’s test (*P* = 0.558).

**Table 4:** Comparison of three preoperative factors (cBMI, mBMI, and albumin) by ROC curve.

Variable		AUC	95% Confidence Interval	
			Lower Limit	Upper Limit
cBMI Model	Not adjusted	0.659	0.648	0.670
	Adjusted* (1)	0.853	0.846	0.860
mBMI Model	Not adjusted	0.797	0.787	0.807
	Adjusted* (2)	0.879	0.873	0.886
Albumin Model	Not adjusted	0.811	0.802	0.820
	Adjusted* (3)	0.878	0.872	0.885
Overall Model**	Not adjusted	0.820	0.811	0.829
	Adjusted* (4)	0.885	0.878	0.891

^*^Model: adjusted covariates (type of operation, gender, age, history of diabetes mellitus, hypertension, ischemic heart disease, neurologic disease, American society of anesthesiologists classification, history of general anesthesia, type of anesthesia, postoperative intensive care unit admission).

**Overall Model: cBMI + albumin + interaction (cBMI * albumin). DeLong’s test for two ROC curve, (1) vs (4): Z = 16.673, *P* < 0.001, (2) vs (4): Z = 8.753, *P* < 0.001, (3) vs (4): Z = 6.670, *P* < 0.001, (2) vs (3): Z = 0.586, *P* = 0.558.

## Discussion

Our study showed that low cBMI, mBMI, and albumin are risk factors of postoperative 1-year mortality; however, when considering the interaction between cBMI and albumin, albumin was the only significant factor of postoperative 1-year mortality. Furthermore, adding cBMI to albumin does not increase the validity of the AUC of albumin in predicting postoperative 1-year mortality. These findings are important and meaningful, because they were obtained from the general population of more than 80,000 patients over five years at a 1,360-bed tertiary care hospital. Furthermore, this is the first study that used mBMI in general population cohorts; mBMI has been previously used for liver transplantation patients.

The first thing to consider when interpreting our findings is the superiority between serum albumin and cBMI in representing patients’ malnutrition status. A previous study reported that for patients on dialysis, albumin was an independent risk factor of operative mortality or morbidity, while cBMI was not^3^. In contrast, low cBMI was associated with mortality in patients undergoing cardiac surgery, while serum albumin was not^14^. Therefore, it is imperative to identify the index with a greater sensitivity for postoperative prognosis. We verified that albumin is a better index than cBMI in predicting postoperative prognosis. The reason for this superiority of albumin is that it reflects protein energy malnutrition unlike cBMI, which simply reflects the nutritional status of patients^15^. Low serum albumin is a measure of exacerbated immunity^16^ and a risk factor of increased postoperative infection^17,18^. Therefore, serum albumin is a better predictor of postoperative prognosis than cBMI.

We showed that mBMI has an equal predictive power to serum albumin with postoperative 1-year mortality. In addition, when interactions between albumin and cBMI were considered, albumin was the only independent predictor of postoperative 1-year mortality. It means that serum albumin was the most important factor for predicting 1-year mortality rather than cBMI. Previous studies have hypothesised that mBMI would be superior to cBMI in predicting mortality because it also measures the effects of fluid accumulation; therefore, mBMI would be more important for patients with ascites or edema^2,10^. Similarly, our study showed that mBMI is a stronger predictor of postoperative mortality is than cBMI in postoperative patients. We hypothesised that mBMI would be better than cBMI and serum albumin because mBMI has the characteristics of cBMI and albumin used in predicting postoperative prognosis. However, our study showed that low mBMI was not a better indicator of postoperative 1-year mortality than serum albumin, and adding cBMI to albumin does not increase the validity of the AUC of albumin in predicting postoperative 1-year mortality. Furthermore, mBMI was also not an independent risk factor when considering the interactions among variables. Based on this, we suggest that mBMI is a more appropriate index than only cBMI for use in patients undergoing surgery. Moreover, using mBMI is not recommended for predicting postoperative 1-year mortality rather than serum albumin; because serum albumin is the strongest index for predicting 1-year mortality after surgery.

In addition, there is an important consideration for using mBMI; unlike albumin levels or cBMI, which are currently the standard indices, there are no clear classification criteria for the normal ranges of mBMI. Tanaka *et al*. classified mBMI into 6 groups (<600, 600–800, 800–1000, 1000–1200, 1200–1400, and >1400)^11^. Suhr *et al*. reported that mortality was high in the liver transplantation group with mBMI <600^12^. However, no other study has classified mBMI more appropriately and investigated its association with mortality. Since mBMI involves a wider range of measurements (600–1400) than albumin or cBMI, the OR of mBMI seems smaller than that of cBMI or albumin. Considering this wide range of mBMI, further studies are warranted to identify appropriate classifications of mBMI for its clinical application.

Our study has a few limitations. First, the retrospective design may have led to selection or detection bias. However, this analysis based on all general surgical adult populations, included a large sample size of more than 80,000.

Second, the generalizability of our findings is limited because we reviewed the medical records at a single center. Third, serum albumin levels were checked at different time points across patients, so there may be differences in the measurements according to the disease severity. Finally, we could not take into consideration the surgical techniques used and postoperative care during the study period of 5 years. Nevertheless, our study is meaningful in that it is the first study that compared the association of mBMI, albumin, and cBMI with 1-year mortality in general surgical patients.

In conclusion, our study showed that low albumin was the strongest and independent risk factor of postoperative 1-year mortality compared to cBMI or mBMI. Adding cBMI to albumin does not increase the validity of the AUC of albumin in predicting postoperative 1-year mortality.

## Methods

This study was a retrospective observational study and was approved by the Institutional Review Board at the SNUBH (Approval Number: B1705/395-106). The requirement for written informed consent was waived by the IRB, and this manuscript adheres to the applicable STROBE guidelines. The medical records of adult patients who were admitted to the SNUBH between January 2011 and December 2015 and underwent an elective or emergency surgical procedure were collected. Patients with inaccurate or incomplete medical records were excluded from the analysis. When one patient underwent two or more surgeries, only the medical record for the final surgery was included. The SNUBH is a 1,360-bed tertiary care academic hospital, where about 150 elective or emergency surgical operations are performed in 38 operating rooms on average every day. Furthermore, the hospital has been keeping and managing medical records since 2003 using an electronic medical system.

### Definition of mBMI

We defined and calculated mBMI using the method suggested by a previous study^10,11^. The mBMI was calculated by multiplying cBMI (kg/m^2^) with preoperative serum albumin level (g/L). We used heights and weights taken before the surgery or at the time of admission and serum albumin level determined at the day closest to the surgery and at least 4 weeks prior to the surgery.

### Data Collection and outcome

The following medical records were collected for the study: gender, age (year), height (cm), weight (kg), cBMI (kg/m^2^), preoperative blood laboratory test results [haemoglobin (g/dL), white blood cell count (*1000/µL), platelet count (*1000/µL), prothrombin time (INR), aspartate aminotransferase (IU/L), alanine aminotransferase (IU/L), glucose (mg/dL), activated partial thromboplastic time (sec), blood urea nitrogen (mg/dL), creatinine (mg/dL), sodium (mmol/L), potassium (mmol/L)], Cormack grade, [history of diabetes mellitus, hypertension, ischaemic heart disease, and neurologic disease], American society of anesthesiologists (ASA) classification, history of general anesthesia, type of operation and anesthesia, postoperative ICU admission, and death date.

Only preoperative blood laboratory test results obtained within 1 month before the surgery were used. The type of operation was classified into cardiovascular surgery and non-cardiovascular surgery. Cardiovascular surgery was defined as cardiac surgery or major vascular surgery involving cardiopulmonary bypass. All medical records were collected by a medical record technician of the SNUBH’s medical informatics team, who was blinded from the aims of this study; the main researchers were also blinded from the data until the final statistical outcomes were available. Moreover, the accurate dates of death of all patients (as of July 1, 2017) were obtained with approval from the Ministry of the Interior and Safety in Korea.

The primary outcome evaluated was the value of preoperative mBMI as a prognostic factor for postoperative 1-year mortality. Therefore, we compared mBMI with cBMI and albumin levels.

### Statistical Methods

The baseline characteristics of all patients are presented as percentage (%) or mean and standard deviation. Continuous variables were analysed using the *t*-test and categorical variables were analysed using the chi-square test to evaluate the association of each variable with 1-year survival or death. The risk for postoperative 1-year mortality with respect to the three variables (cBMI, mBMI, and albumin) was analysed using restricted cubic spline, and the odds ratios (ORs) for postoperative 1-year mortality for each variable were computed using univariate and multivariate logistic regression analysis. We performed univariate logistic regression analysis to determine factors associated with postoperative 1-year mortality individually. We then selected covariates significant at *P* < 0.05 in the univariate logistic model to be included in the multivariate logistic regression analysis. At multivariate logistic regression analysis, we built two models considering the interaction among cBMI, mBMI, and albumin. Finally, the power of each of the three variables (cBMI, mBMI, and albumin) for explaining postoperative 1-year mortality was computed using the receiver operating characteristic (ROC) curve; areas under the curve (AUC) were compared and tested using the DeLong’s test. Additionally, we used ROC curves, after adjusting for the covariates that affected postoperative 1-year mortality, to compute adjusted AUC for each variable. The covariates adjusted for in the AUC were selected from the univariate logistic regression analysis based on the criterion of *P* < 0.05.

The power of the sample size of our study was verified by the PASS 15 program. From the area under the curve (AUC) of 0.8, to detect the difference of 0.05 of the AUC from the event rate of 4.4%, 15,000 patients were adequate to yield 80% power and an alpha error of 0.05. Therefore, our study was determined to have sufficient power to detect a difference of 0.05 from an AUC of 0.8 in predicting 1-year mortality. Statistical analyses were performed using IBM SPSS (Version 23.0; IBM Corp., Armonk, NY, USA) and R (Version 3.3.2 with R packages; http://www.R-project.org) software, with statistical significance set at *P* < 0.05.

### Data availability

The datasets generated during the current study are available from the corresponding author on reasonable request.

## Electronic supplementary material

Supplementary Table 1
