# Supplementary material for: Value of Preoperative Modified Body Mass Index in Predicting Postoperative 1-Year Mortality
Source: Sci Rep. 2018 Mar 15;8:4614. doi: 10.1038/s41598-018-22886-6 (PMC5854572; doi:10.1038/s41598-018-22886-6)
Supplement: Supplementary file 1 — Supplementary Table 1 [file 41598_2018_22886_MOESM1_ESM.pdf]

Value of Preoperative Modified Body Mass Index in Predicting Postoperative 1-Year Mortality

Tak Kyu Oh[1,2], Jaebong Lee[3] , Jung-Won Hwang[2], Sang-Hwan Do[2], Young-Tae Jeon[2], Jin Hee Kim[2], Kooknam Kim[2], In-Ae Song[1,2*]

[1] Interdepartment of Critical Care Medicine, Seoul National University Bundang Hospital, 82, Gumi-ro 173 Beon-gil, Bundang-gu, Seongnam-si, Gyeonggi-do, Korea 463-707

[2] Department of Anesthesiology and Pain Medicine, Seoul National University Bundang Hospital, 82, Gumi-ro 173 Beon-gil, Bundang-gu, Seongnam-si, Gyeonggi-do, Korea 463-707

[3] Medical Research Collaborating Center, Seoul National University Bundang Hospital, 82, Gumi-ro 173 Beon-gil, Bundang-gu, Seongnam-si, Gyeonggi-do, Korea 463-707

Correspondence and reprint requests:

*In-Ae Song, MD, PhD

Department of Anesthesiology and Pain Medicine, Seoul National University Bundang Hospital, 166, Gumi-ro, Bundang-gu, Seongnam 463-707, Korea

Tel: 82-31-787-7499, Fax: 82-31-787-4063, Email: songoficu@outlook.kr

Supplemental table 1. Multivariate logistic regression analysis for one-year mortality regarding three preoperative variables (cBMI, mBMI, and albumin)

	(+) cBMI		(+) mBMI		(+) Albumin	
Variable	Odds Ratio (95% CI)	*P*-value	Odds Ratio (95% CI)	*P*-value	Odds Ratio (95% CI)	*P*-value
Type of operation						
Non-cardiovascular surgery	ref		ref		ref	
Cardiovascular surgery	0.631 (0.498-0.801)	<0.001	0.688 (0.540-0.877)	0.003	0.634 (0.498-0.808)	<0.001
Gender						
Male	ref		ref		ref	
Female	0.539 (0.494-0.588)	<0.001	0.540 (0.494-0.591)	<0.001	0.538 (0.492-0.588)	<0.001
Age (yr)	1.041 (1.038-1.045)	<0.001	1.032 (1.028-1.036)	<0.001	1.035 (1.031-1.038)	<0.001
cBMI (kg/m^2)	**0.847** (0.836-0.858)	<0.001				
mBMI			**0.995** (0.995-0.995)	<0.001		
Albumin (g/dl)					**0.230** (0.214-0.247)	<0.001
Diabetes mellitus						
No	ref		ref		ref	
Yes	1.079 (0.969-1.203)	0.167	1.063 (0.950-1.190)	0.285	0.978 (0.873-1.094)	0.693
Hypertension						
No	ref		ref		ref	
Yes	0.635 (0.576-0.701)	<0.001	0.732 (0.662-0.811)	<0.001	0.612 (0.554-0.677)	<0.001

History of IHD						
No	ref		ref		ref	
Yes	0.757 (0.658-0.871)	<0.001	0.839 (0.726-0.969)	0.017	0.843 (0.730-0.973)	0.019
History of NUD						
No	ref		ref		ref	
Yes	0.964 (0.829-1.122)	0.639	0.954 (0.817-1.114)	0.552	1.022 (0.876-1.193)	0.782
ASA class						
I	ref		ref		ref	
II	3.714 (3.211-4.295)	<0.001	3.052 (2.634-3.537)	<0.001	2.749 (2.372-3.186)	<0.001
III	11.796 (9.915-14.034)	<0.001	6.454 (5.393-7.722)	<0.001	5.651 (4.716-6.772)	<0.001
IV, V, VI	31.919 (21.579-47.215)	<0.001	13.959 (9.238-21.091)	<0.001	10.594 (6.970-16.102)	<0.001
History of general anesthesia						
No	ref		ref		ref	
Yes	1.408 (1.292-1.534)	<0.001	1.305 (1.194-1.425)	<0.001	1.270 (1.162-1.387)	<0.001
Type of anesthesia						
General anesthesia	ref		ref		ref	
Regional anesthesia	0.600 (0.516-0.697)	<0.001	0.550 (0.472-0.641)	<0.001	0.495 (0.425-0.576)	<0.001
Monitored anesthesia care	1.125 (1.015-1.245)	0.024	1.086 (0.978-1.207)	0.124	1.140 (1.026-1.267)	0.015
Local anesthesia	3.446 (1.003-11.841)	0.049	2.760 (0.738-10.330)	0.132	3.226 (0.868-11.991)	0.080
Postoperative ICU admission						
No	Ref		ref		ref	

Yes	2.103 (1.825-2.422)	<0.001	1.680 (1.452-1.943)	<0.001	1.728 (1.493-2.000)	<0.001

cBMI, Conventional Body Mass Index; mBMI, modified Body Mass Index; IHD, Ischemic Heart Disease; NUD, Neurologic Disease; ASA, American Society of Anesthesiologists; ICU, Intensive Care Unit
